# Drought induced metabolic shifts and water loss mechanisms in canola: role of cysteine, phenylalanine and aspartic acid

**DOI:** 10.3389/fpls.2024.1385414

**Published:** 2024-12-23

**Authors:** Raed Elferjani, Shankar Pahari, Raju Soolanayakanahally, Krista Ballantyne, Eiji Nambara

**Affiliations:** ^1^ Saskatoon Research and Development Centre, Agriculture and Agri-Food Canada, Saskatoon, SK, Canada; ^2^ Department of Cell and Systems Biology, University of Toronto, Toronto, ON, Canada

**Keywords:** *Brassica napus*, drought, amino acids, cysteine, phenylalanine, aspartic acid, ABA, epicuticular waxes

## Abstract

Drought conditions severely curtail the ability of plants to accumulate biomass due to the closure of stomata and the decrease of photosynthetic assimilation rate. Additionally, there is a shift in the plant’s metabolic processes toward the production of metabolites that offer protection and aid in osmoadaptation, as opposed to those required for development and growth. To limit water loss via non-stomatal transpiration, plants adjust the load and composition of cuticle waxes, which act as an additional barrier. This study investigates the impact of soil water deficit on stomatal and epicuticular water losses, as well as metabolic adjustments in two canola (*Brassica napus* L.) cultivars—one drought-tolerant and the other drought-sensitive. Specifically, we examined the effect of a drought treatment, which involved reducing water holding capacity to 40%, on the levels of cysteine, sucrose, and abscisic acid (ABA) in the leaves of both cultivars. Next, we looked for potential differences in night, predawn, and early morning transpiration rates and the epicuticular wax load and composition in response to drought. A substantial rise in leaf cysteine was observed in both canola cultivars in response to drought, and a strong correlation was found between cysteine, ABA, and stomatal conductance, indicating that cysteine and sulfur may play a role in controlling stomatal movement during drought stress. Attributes related to CO_2_ diffusion (stomatal and mesophyll conductance) and photosynthetic capacity were different between the two canola cultivars suggesting a better management of water relations under stress by the drought-tolerant cultivar. Epicuticular waxes were found to adjust in response to drought, acting as an additional barrier against water loss. Surprisingly, both canola cultivars responded similarly to the metabolites (cysteine, sucrose, and ABA) and epicuticular waxes, indicating that they were not reliable stress markers in our test setup. However, the higher level of phenylalanine in the drought-tolerant canola cultivar is suggestive that this amino acid is important for adaptation to drier climates. Furthermore, a multitrait genotype-ideotype distance index (MGIDI) revealed the likely role of aspartic acid in sustaining nitrogen and carbon for immediate photosynthetic resumption after drought episodes. In conclusion, leveraging amino acid knowledge in agriculture can enhance crop yield and bolster resistance to environmental challenges.

## Introduction

Soil water deficit is one of the most significant plant abiotic stressors, which leads to famines and food shortages across the world, resulting in considerable income loss for farmers (Li et al., 2009). To counterbalance the productivity losses of crops due to episodic or prolonged drought, substantial research has examined the effect of abiotic stressors on two major processes: i) shifts in plant metabolites and the subsequent effect on carbon assimilation and growth, and ii) transpirational water loss through stomata and non-stomata via leaf cuticle. Since water loss by transpiration shares the same pathways (i.e., stomata) with carbon dioxide entry, reducing water loss by transpiration has significant impacts on carbon assimilation and biomass production (Iqbal et al., 2020). Furthermore, drought episodes have an impact on both day and night temperatures, which in turn affects transpiration rates (Sadok and Tamang, 2019). Observing the water transpiration and carbon assimilation rates of plants during the day and night might help us understand how plants cope with drought. With this information, we can gain a better understanding of how crops manage stomata aperture during seasonal circadian cycles to maximize water-use efficiency (WUE) without compromising yields.

It may be possible to use metabolites as markers that are highly responsive to stressors to develop stress-tolerant varieties and hybrids by conventional or molecular genetic improvement (Sreeman et al., 2018; Salami et al., 2024a). Some metabolites have been reported as species-specific stress markers and are being used in breeding programs to develop stress-tolerant cultivars (Hill et al., 2015; Chaitanya et al., 2024). An example of a stress indicator is cysteine, the first product of sulfate assimilation. Many studies reported the role of cysteine as a signaling and scavenging molecule in antioxidant defense (Hasanuzzaman et al., 2018; Jobe et al., 2019). The cysteine protease, along with other proteases, is involved in stress-induced proteolysis in plants, where stress-altered proteins are degraded to become non-functional and trigger leaf senescence and plant reproduction through protein turnover (Fenta et al., 2012; Kidrič et al., 2014; Quain et al., 2014; Botha et al., 2017). Besides its role in cysteine synthesis and as an antioxidation component, sulfur (in particular, hydrogen sulfide [H_2_S]), is also a signaling molecule that plays an important role in regulating the movement of guard cells of stomata in response to water deficit and other stimuli (Raghavendra et al., 2010; Pantaleno et al., 2021). Sucrose, the main product of photosynthetic activity and source of energy for plants, is also a signaling molecule for growth and is associated with the tolerance to abiotic stress as a compatible solute (Du et al., 2020). The transport of sucrose and carbohydrate metabolism are responsive to environmental cues and have been used to assess drought and salinity tolerance (Muller et al., 2011). Furthermore, stress signaling molecules such as ABA were shown to have a role in sucrose transport and distribution for osmoregulation (Mathan et al., 2021).

On leaves and fruits of many crops, epicuticle waxes (EWs), which are long-chain fatty acid derivatives, have been extensively investigated for their role as pest barriers (especially thrips). Many studies have investigated the possibility of EW accumulation and composition conferring drought resistance in crops, with some promising results reported (Seo and Park, 2011; Tassone et al., 2016; Zhong et al., 2020). Tassone et al. (2016) found that 24 leaf cuticular chemical components were highly heritable among 517 *Brassica napus* accessions, with C29 alkanes in particular being recognized as possible stress tolerance breeding traits. Nevertheless, EW accumulation and composition vary quite significantly between species and stress conditions, which requires species-specific research to assess the relative role of EW variation in drought tolerance (Xue et al., 2017).

Canola (*Brassica napus* L.) is a major oilseed crop with a global production estimated to be 88.34 million metric tons for 2024 (U.S. Department of Agriculture, 2024). Canola oil which is rich in healthy polyunsaturated fatty acids, is the third most used oil for the world food industry. However, canola is subject to loss of yield due to more frequent episodes of drought which might compromise world supply. Many studies on gene discovery and crop improvement are attempting to maintain productivity under water deficit conditions by using metabolic, morphological, and physiological markers of drought tolerance (Salami et al., 2024b; Tassone et al., 2016). The purpose of this study was to compare stomatal and epicuticular water losses and metabolic responses to the soil water deficit between drought-tolerant and drought-sensitive canola cultivars. We examined the amino acid cysteine to understand how it affects stomatal movement and photosynthetic activity in response to drought. It was hypothesized that cysteine might reduce water losses by modulating stomata closure and accumulation of leaf epicuticular waxes in canola, thereby ameliorating drought mitigation. Next, we shed light on the role of phenylalanine, aspartic acid, and serine as a “dynamic trio” in improving canola’s ability to withstand intermittent drought conditions.

## Materials and methods

### Growth environment

In this greenhouse study, two spring canola cultivars previously screened for drought tolerance were utilized: the drought-tolerant cultivar “Czyzowska” and the drought-sensitive cultivar “BN-1.” These cultivars served as founder lines for developing a nested association mapping population (Ebersbach et al., 2022). For each cultivar, six tubs each with 60 L capacity were filled with peat moss + soil mix and watered to field capacity. The bottom was perforated (six holes) to allow water drainage. A slow-releasing fertilizer (Osmocote, Everris, USA) was added at 10.7 g L^−1^ to avoid nutrient deficiencies. Nine canola seeds per tub were sown at an equal distance and thinned after emergence to five plants per tub. Plants were grown under a day/night temperature regime of 23°C/18°C, respectively, and relative humidity of 45% to 65%. The day/night photoperiod was set at 16 h/8 h with a minimum photosynthetic photon flux density (PPFD) of at least 400 μmol m^−2^ s^−1^ during the day supplemented by sunlight. Plants were regularly watered until bolting. From bolting to physiological maturity, the temperature was increased from 23°C to 28°C over a 60-min period starting at 09:30 h and stayed at 28°C till 15:30 h to ramp down to 23°C within a 60-min window.

Once plants started bolting, tubs were randomly assigned to one of the two treatments (*N* = 12: 2 treatments × 2 cultivars × 3 replications)—i) control treatment (WW): to mimic field conditions, plants were watered at 90% of maximum water holding capacity (WHC), and ii) drought treatment (D): plants were exposed to 40% of WHC. The water holding capacity (%) of the growth medium was determined as described in Elferjani and Soolanayakanahally (2018). Prior to physiological maturity, plants exposed to drought were watered optimally (90% of WHC) and allowed 48-h recovery before leaf sampling for amino acid analysis.

### Growth and yield

Leaf temperature was monitored with a thermal imaging camera (FLIR T530, FLIR Systems, Wilsonville, Oregon, USA) by taking images of the canopy at 08:00 h, 12:00 h, 14:00 h, 16:00 h, and 18:00 h. The normalized difference vegetation index (NDVI) was measured using a GreenSeeker handheld crop sensor (Trimble, Westminster, CO, USA). The sensor was held 80 cm above the plant canopy, as recommended by the manufacturer. The measurements were taken between 09:00 h and 11:00 h. Plants were harvested at maturity and pods were collected in brown paper bags and stored under ambient temperature for 3–5 days until threshing. Seeds were cleaned and weighed, and oil and protein contents were determined using the NIR method as described in Siemens and Daun (2005).

### Whole plant transpiration

In a separate experiment, the canola cultivars (Czyzowska and BN-1) were grown in 2-L pots (1 plant per pot) filled with peat moss soil mix and regularly watered under 23°C/18°C and 18 h/6 h day/night temperature and photoperiod (*N* = 10: 5 replicates × 2 cultivars). At the four-leaf stage, water loss by transpiration of plants was monitored by weighing pots, using an electronic balance. First, plants were watered to field capacity, then medium surface and pot edges were covered with aluminum foil to prevent water evaporation from the substrate, and the initial weight of each pot was recorded at 19:00 h (i.e., greenhouse lights were off). Then, pots were weighed every 2 h for the next 36 h. After pot weighing, the second fully developed leaf from the top of each plant was sampled and petiole tips were covered with Vaseline to assess non-stomatal transpiration. Cut leaves were immediately transferred to a dark room at 23°C to minimize stomatal water loss, where their initial weight and surface area were measured. Then, their weights were recorded every 30 min from 10:00 h to 18:00 h.

### Photosynthetic activity

The portable photosynthesis LI-6400XT system equipped with a 6400-08 chamber attached to a 6400-02B LED light source (LICOR Inc., Lincoln, NE, USA) was used to measure leaf gas exchanges on the 12th to 15th days following the start of the stress treatment. Measurements were made on the 4th fully developed leaf from the top (*N* = 12: 2 treatments × 2 cultivars × 3 replicates) between 10:30 h and 12:30 h. The response of the net photosynthesis (*A*, μmol m^−2^ s^−1^) to the changing *C*_i_ was measured under saturated photon flux density, PPFD = 1,000 μmol m^−2^ s^−1^. The leaf was first exposed to the ambient atmospheric CO_2_ concentration, *C*_a_ (400 μmol CO_2_ mol^−1^) in the chamber using CO_2_ cartridges to reach a steady state. Next, *C*_a_ was changed in the following order: 400, 300, 200, 100, 50, 400, 500, 600, 800, 1,000 and 1,200 μmol mol^−1^. At each step, we ascertained that the net photosynthetic assimilation rate (*A*), water vapor, and CO_2_ fractions reached steady values at each step before moving to the next step. During the measurement periods, the leaf chamber temperature was set to the ambient temperature (28°C), airflow at 500 μmol s^−1^, relative humidity at 55–65%, and vapor pressure deficit (VPD) at 1.4 ± 0.2 kPa. The order of the measurements was randomized among the treatments and the cultivars throughout the measuring period. *A* (μmol CO_2_ m^−2^ s^−1^) and *g*_s_ (mol CO_2_ m^−2^ s^−1^) values were extracted from *A*–*C*_i_ response measurements for *C*_a_ = 400 μmol CO_2_ mol^−1^. The intrinsic water-use efficiency was then deduced (*A*/*g*_s_). The maximum rate of RuBisCO carboxylation (*V*_cmax_, µmol m^−2^ s^−1^), the rate of photochemical electron transport (*J*, µmol e^−^ m^−2^ s^−1^), and the rate of CO_2_ diffusion through the leaf mesophyll conductance (*g*_m_, µmol m^−2^ s^−1^) were estimated by *A*–*C*_i_ curve fitting, according to Ethier and Livingston (2004); Ethier et al. (2006), and the biochemical model of C_3_ photosynthesis developed by Farquhar et al. (1980) as detailed in Elferjani and Soolanayakanahally (2018).

### Night gas exchanges

The same leaves were subjected to gas exchange measurements at night 16 days after the stress started, using the same apparatus used to measure photosynthetic activity during the day. Leaf chamber parameters that were changed were as follows: PPFD = 0 μmol m^−2^ s^−1^, leaf temperature = 18°C, and airflow = 200 μmol s^−1^. While the *C*_a_ was maintained at 400 μmol CO_2_ mol^−1^ as well as *A*, VPD and CO_2_ fractions were allowed to stabilize before records could be taken. Leaf gas exchanges were monitored twice during the night: the first measurement at night (between 21:45 h and 22:45 h) and the second measurement at predawn (between 03:00 h and 04:00 h). Daytime gas exchanges were also measured early in the morning (between 05:00 h and 06:00 h) with PPFD = 1,000 μmol m^−2^ s^−1^, airflow = 500 μmol s^−1^, and leaf temperature set at 23°C.

### Leaf epicuticular waxes

One fully expanded leaf was sampled from both cultivars (*N* = 24: 2 cultivars × 2 treatments × 3 replicates × 2 leaf surfaces) immediately after gas exchange measurements. Freshly cut leaves were kept in a cool and dry container and stored in a refrigerator at 4°C before being analyzed the next day at the Canadian Light Source Facility (https://www.lightsource.ca/Saskatoon, SK, Canada). Mid-infrared (mid-IR) spectroscopy was used to determine the total wax load and to identify the different functional groups of the epicuticular wax on canola leaves according to the protocol of Willick et al. (2018). Briefly, the mid-IR attenuated total internal reflection (ATR) spectra of fresh leaves were collected using the Cary 600 series FTIR spectrometer (Agilent Technologies, Santa Clara, CA, USA). The ATR crystal used was germanium (45 degrees). Mid-IR data in the spectral range between 4,000 and 600 cm^−1^ (wave numbers) at a resolution of 4 cm^−1^ were recorded at 256 scans per sample on average. Three parameters were considered to characterize the variation of functional groups of waxes following the application of treatments—i) aromatic carbon groups (C=C functional group, 1,650–1,500 cm^−1^): spectral region with variable intensity depending on the plant species (Heredia-Guerrero et al., 2014); ii) methylene/methyl ratio (CH_2_/CH_3_, 3,000–2,800 cm^−1^): related to the length of the aliphatic chain and to the branching of the side groups. A higher value of this ratio indicates longer and straighter (less branched) chains; iii) carbonyl (C=O, 1,800–1,600 cm^−1^): the relative contribution of carbonyl/carboxyl group to a group containing oxygen + aromatic carbon (C=C) structures.

### Scanning electron microscopy of the leaves

Samples were prepared for scanning electron microscopy (SEM) by sampling a 5 × 5-mm portion of the leaf, taped to aluminum foil, and allowed to air dry for 1 week in a desiccator. Samples were then mounted on 9 mm diameter aluminum specimen holders with double-sided carbon tape. Mounted samples were Au sputter-coated in a Denton Vacuum Desk IV Sputtering unit. Sputtering utilized air for plasma gas at 50 mTorr; 38% power applied to the target resulted in 10 mA of current achieving 26 nm film thickness at a 4.3 nm/s deposition rate. SEM was conducted using a Zeiss EVO 60 at 10.19 kV and 8 mm working distance. Three images at different magnifications were collected at ×125, ×2,000 and ×5,000 with 895.2 nm, 55.84 nm, and 22.33 nm spatial resolutions, respectively.

### Stomata metrics

Fully developed leaves were sampled to assess the stomatal density on the abaxial and the adaxial sides using the clear varnish and tape technique. Leaves were coated with a clear varnish and allowed to dry for approximately 1 min. Then, the clear tape was applied on the varnished surface, then peeled off and stuck onto a glass slide. The number of stomata of the epidermal impression on each slide was counted using a fluorescence microscope (Axio Imager Z1, ZEISS, Oberkochen, Germany) and ZEN software (2.3 Pro, ZEISS). Stomatal density, pore length, and pore width were calculated using the scale tool of the ZEN software.

### Abscisic acid content

Fully expanded leaves (third or fourth leaf from the top) were harvested and immediately packed in plastic tubes and frozen in liquid nitrogen before being stored in a −80°C freezer until processed for analyses. Abscisic acid content was determined as previously described (Yan et al., 2016). Briefly, the freeze-dried and powdered samples were suspended in methanol containing 1% acetic acid, and d6-ABA was added as an internal standard and placed in the fridge overnight. The samples were centrifuged to remove debris, and the pellet was washed twice. The supernatant was evaporated in a SpeedVac and reconstituted in 1 mL of 1% (v/v) acetic acid. ABA was purified by solid-phase extraction using Oasis HLB, MCX, and WAX cartridge columns (Waters). The solvent was removed under vacuum and subjected to LC-ESI-MS/MS analysis (Agilent 6,410 Triple Quad LC/MS system). An LC (Agilent 1200 series) equipped with a 50 × 2.1-mm, 1.8-μm Zorbax SB-Phenyl column (Agilent) was used with a binary solvent system comprising 0.01% (v/v) acetic acid in water (solvent A) and 0.05% (v/v) acetic acid in acetonitrile (solvent B). Separations were performed using a gradient of increasing acetonitrile content with a flow rate of 0.2 mL min^−1^. The gradient was increased linearly from 3% B to 50% B over 15 min. The retention time of ABA was 14.0 min. MS/MS transitions 269/159 (d6-ABA) and 263/153 (ABA) were used to quantify ABA.

### Amino acid and sucrose analysis

Total amino acids were extracted from 10 mg powder of freeze-dried tissue samples following Inaba et al. (1994) with some modifications. Briefly, 1.5 mL of 80% (v/v) ethanol solution was added to each sample and shaken for 30 min at 40°C, and the supernatant was recovered by centrifugation (4,000 rpm for 10 min) at 4°C. Amino acids were derivatized following the Waters AccQ-Tag Reagent Kit (Waters, Milford, Massachusetts, USA; Cohen and Michaud, 1993). Briefly; 10 µL aliquot of the sample was mixed with 70 µL of borate buffer and 20 µL of AccQ-Fluor reagent which was reconstituted in acetonitrile. The AccQ-Fluor reagent was reconstituted as follows: 1 mL of AccQ-Fluor reagent diluent was transferred to a vial containing AccQ-Fluor reagent powder and vortexed for 10 s before heating at 55°C for a maximum of 10 min or until dissolved. The derivatized mixture was transferred to an autosampler vial and incubated at 55°C for 10 min. The derivatized samples were subjected to high-performance liquid chromatography (HPLC) as described in the Waters AccQ-Tag Chemistry Package Instruction manual, with an excitation wavelength of 285 nm and emission wavelength of 320 nm on a Waters Amino Acid Column—3.9 × 150 mm using 2475 scanning fluorescence detector (Waters, Milford, Massachusetts, USA). The column was set at 37°C with 5 µL of injection volume. Waters AccQ-Tag buffer (100 mL of AccQ-Tag Buffer concentrate + 1,000 mL of Super-Q water), acetonitrile, and Super-Q water were used as mobile phase A, mobile phase B, and mobile phase C, respectively. The concentration of amino acids (pmol/µL) from a sample was calculated using peak area values of the chromatogram against the calibration curve of serial dilution (10, 25, 50, 100, 150 pmol/µL) of known amino acid calibration standards (WAT 088122, Waters, Milford, Massachusetts, USA) with α-aminobutyric acid as an internal standard. The values were then converted to µmol/mg using the extraction volume and weight of the initial sample.

Total sugars were extracted from 10 mg powder of freeze-dried leaf tissue samples. One milliliter of 75% (v/v) methanol solution containing 0.1% formic acid was added to each sample and mixed by vortexing for 10 s followed by sonication in a water bath at room temperature for 15 min. The supernatant was obtained by centrifugation (20,000 rpm for 15 min) at room temperature. The resulting supernatants were filtered through a 0.2-μm PVDF filter syringe onto HPLC slit vials and stored at −20°C until use. Sugar analysis was performed on a Waters Acquity UPLC system with evaporative light scattering (ELS) and photodiode array (PDA) detection. A Waters UPLC BEH amide 1.7 μm 2.1 × 100 mm column at 70°C was used with solvents: A—95% ACN/5% water + 0.1% TEA and B—30% ACN/70% water + 0.1% TEA with a 60-min runtime. The ELS detection settings used included gain: 400, mode: cooling, gas pressure: 60.0 psi, and drift tube: 60°C. Sucrose was identified and quantified by comparison with known standards (25 µg/mL) prepared in 80% acetonitrile.

### Statistical analyses

A two-way analysis of variance (ANOVA) was used to test the effects of the cultivar, the water status, and the interaction between them on the measured traits. The means were compared using Tukey’s honest significant difference (HSD) at a *p <*0.05 significance level. The coefficients and the *p*-values of the correlations were calculated using Pearson’s correlation coefficient. All the statistical analyses were performed with R software version 4.2.1 (R Core Team, 2021). A correlation bubble plot was generated using the corrplot function (Taiyun and Viliam, 2017), and bar plots and line plots were generated using the ggplot2 function (Wickham, 2016) in R software. Principal component analysis (PCA) was computed using the online clustVis tool (Metsalu and Vilo, 2015). The multitrait genotype-ideotype distance index (MGIDI) was used to rank the amino acids based on information of multiple traits as proposed by Olivoto and Nardino (2021). Lavaan software package with SEM function (Rosseel, 2012) within the R statistical software (R Core Team, 2021) was used to produce the hypothetical pathway model for observed traits.

## Results

### Agronomic traits

At physiological maturity, plants of the drought-tolerant (DT) canola cultivar were taller and had higher NDVI values than those of the drought-sensitive (DS) cultivar under well-watered (WW) conditions (**Supplementary Figures S1A, B**). The DT cultivar flowered 7 days later (46 days after sowing, DAS) compared to the DS cultivar (39 DAS) under WW conditions (**Supplementary Figure S1C**). Plants grown under drought conditions were shorter in height and flowered earlier (43 DAS and 37 DAS, respectively, for the DT and DS cultivars). Similarly, seed yield and the number of seeds per pod were significantly lower in the DS cultivar compared to the DT cultivar (**Supplementary Figure S1E**). Drought treatment further reduced the yield for both cultivars. The number of branches, however, was higher in the DS cultivar, both of which were reduced under drought conditions (**Supplementary Figure S1D**). On the other hand, the seeds per pod were higher in the DT cultivar (**Supplementary Figure S1F**).

### Diurnal changes in leaf temperature and transpiration water loss

**Figure 1:**
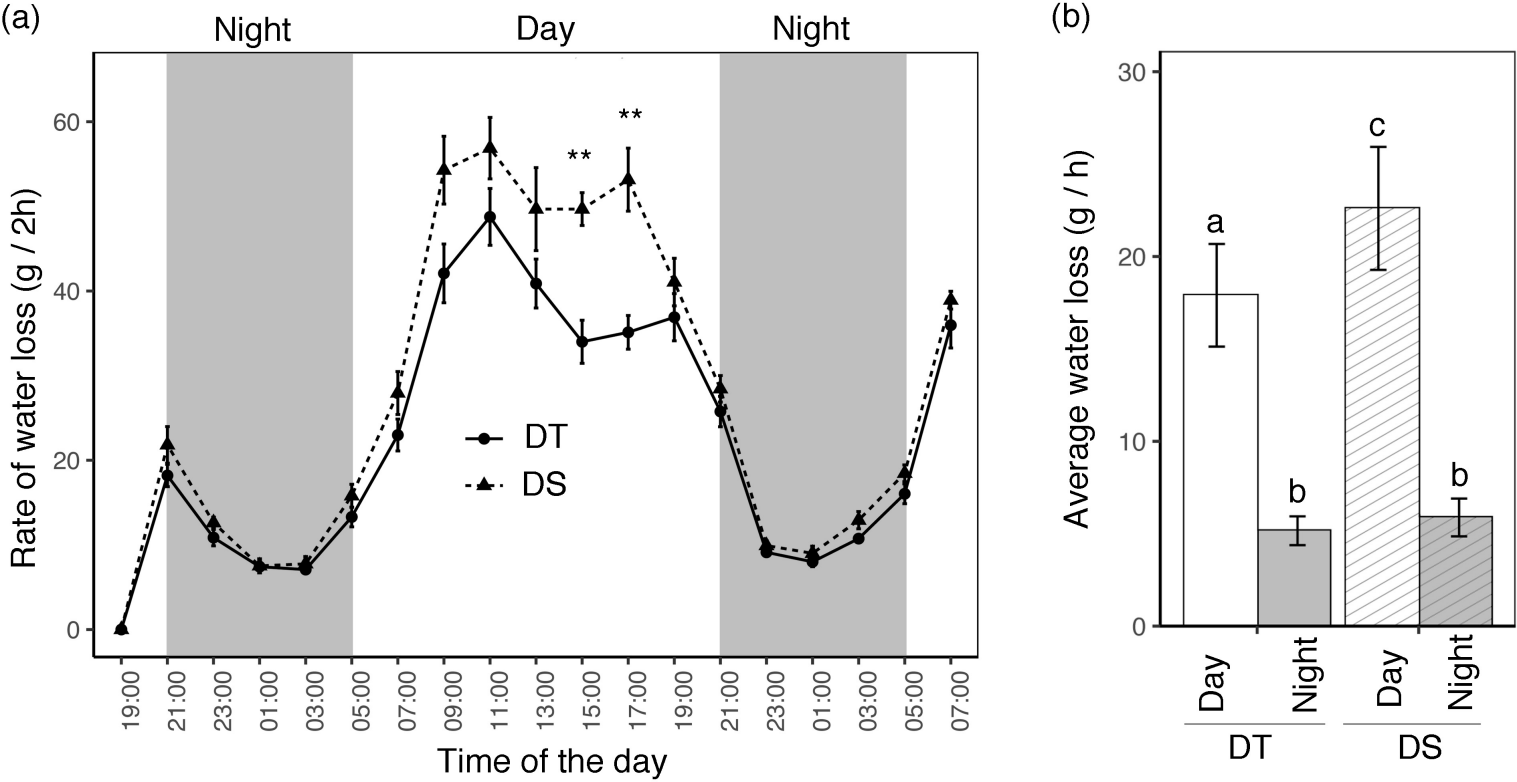
Gravimetrically measured day and night water loss of drought-tolerant (DT) and drought-sensitive (DS) canola cultivars grown in pots. **(A)** The total amount of water lost per pot was measured every 2 h. The gray background represents nighttime (21:00 pm to 5:00 am) and the observations were recorded for two full nights and one full day. Data points represent mean ± SE (*n* = 5 plants). Statistical significance between DT and DS is indicated by asterisks (*p* < 0.01**). **(B)** Day and night average water loss per hour. The statistically significant differences between the cultivars during the day and night are labeled with different letters at *p* <0.05 (Tukey’s HSD).

Leaf temperature was recorded from 08:00 h to 18:00 h every 2 h (**Supplementary Figure S2**). The lowest leaf temperature was recorded at 08:00 h and the highest at 10:00 h when the greenhouse reached the 28°C target temperature. For both the DT and DS canola cultivars, leaf temperature was significantly higher under drought than under WW conditions at every time point. Water loss was measured by weighing every 2 h for two nights and one full day (**Figure 1A**). Based on daylight time and greenhouse conditions, the nighttime period starts at 21:00 h and ends at 05:00 h the next morning. The amount of water lost every 2 h decreased during the night until 03:00, after which it started to rise in the morning, continued to rise throughout the afternoon, and then began to decrease once more during the night. A subtle difference between the two cultivars was observed during the night, but the differences became significant during the day at 15:00 h and 17:00 h (**Figure 1A**). Average water loss per hour was significantly different between DT and DS during the day, but not during the night (**Figure 1B**).

### Leaf stomatal conductance

**Figure 2:**
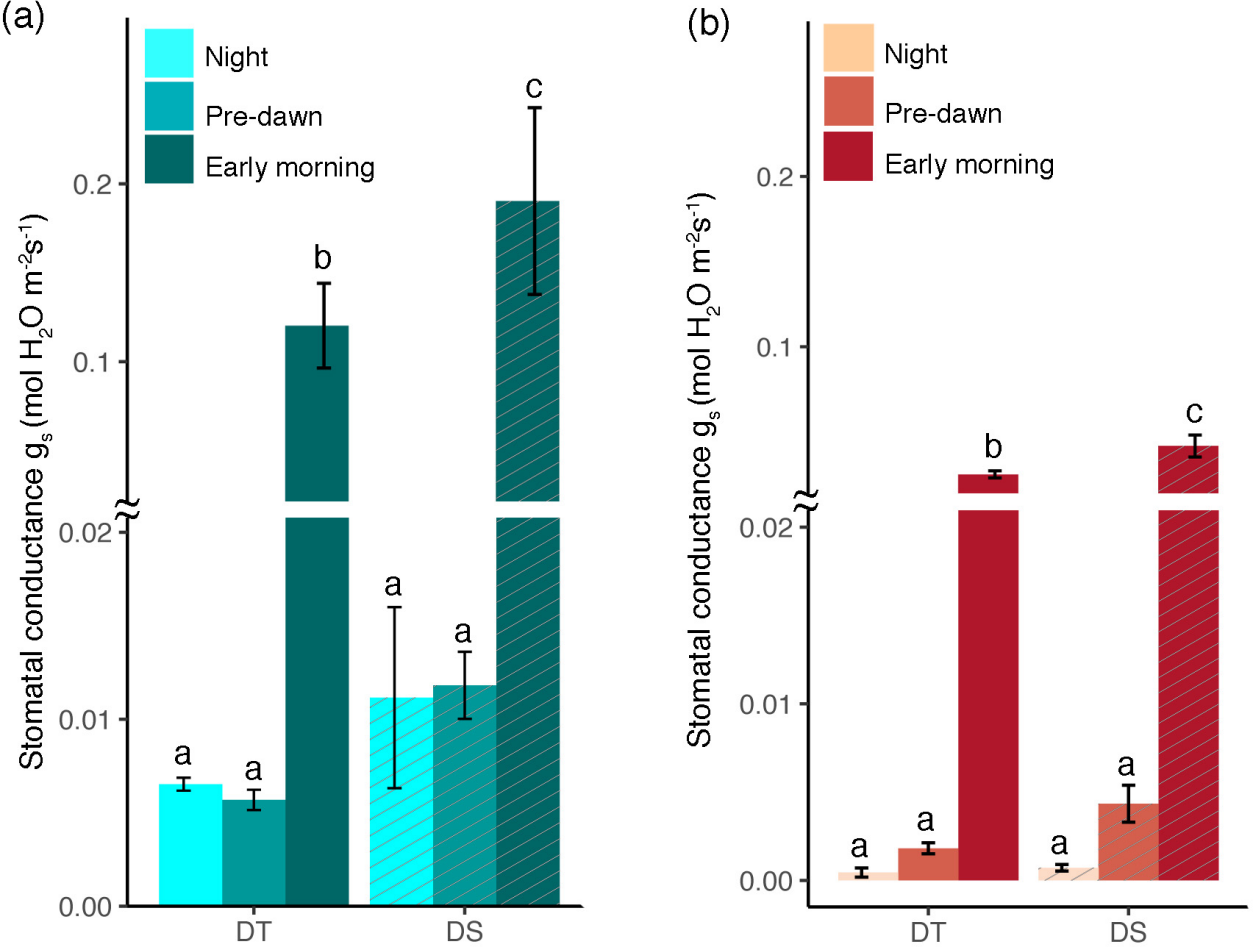
Stomatal conductance (*g*_s_) during the night, predawn, and early morning for the drought-tolerant (DT) and drought-sensitive (DS) canola cultivars under well-watered **(A)** and drought conditions **(B)**. The statistically significant differences between the cultivars at different time points are labeled with different letters at *p* <0.05 (Tukey’s HSD).

**Figure 3:**
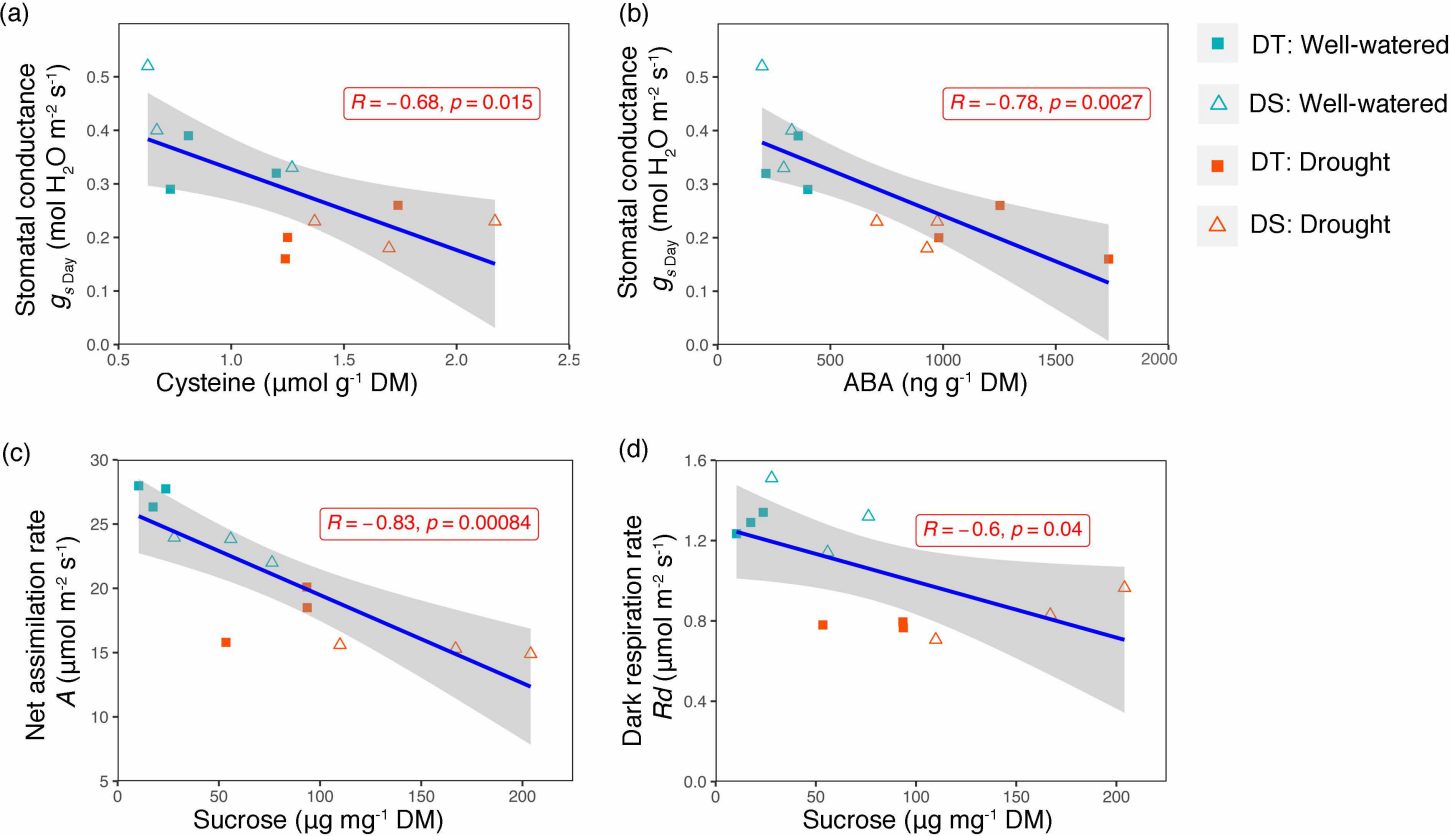
Scatter plot showing linear association between cysteine and stomatal conductance **(A)**, ABA and stomatal conductance **(B)**, sucrose and net assimilation rate **(C)**, and sucrose and dark respiration **(D)**. Pearson correlation coefficient (*R*) and *p*-value for each linear relation are illustrated. The gray shading represents the 95% confidence interval around the line of the best fit.

Leaf stomatal conductance (*g*_s_) was recorded at night, predawn, and in the morning for both the DT and DS canola cultivars under WW and drought conditions. At each of the three time points and under both conditions, *g*_s_ was higher in the DS cultivar (**Figures 2A, B**). Among the time points recorded, there was no significant increase from night to predawn; however, there was a drastic increase during the early morning (**Figures 2A, B**). The early morning stomatal conductance rates under the WW conditions for the DS (0.19 mol m^−2^ s^−1^) and the DT (0.12 mol m^−2^ s^−1^) cultivars were higher than those under drought treatments (0.042 and 0.025 mol m^−2^ s^−1^, respectively, for DS and DT). Stomatal conductance was negatively correlated to leaf cysteine and ABA content (*R* = −0.68, *p* = 0.015 and *R* = −0.78, *p* = 0.0027, respectively) (**Figures 3A, B**). Although ABA content was positively correlated to cysteine (*R* = +0.49, data not shown), the correlation was not significant (*p* = 0.1). Furthermore, sucrose showed a significant negative correlation with net assimilation rate (*R* = −0.83, *p* = 0.00084) and dark respiration rate (*R* = −0.60, *p* = 0.04) (**Figures 3C, D**).

### Relationship between ABA, sucrose, cysteine, and leaf CO_2_ diffusion

Under the WW conditions, leaf ABA content for the DT and DS canola cultivars was similar (323.8 and 273.1 ng g^−1^, respectively). Under drought, ABA content increased significantly to 1,322.7 and 869.7 ng g^−1^ for DT and DS, respectively (**Supplementary Figure S3A**). Sucrose content under the WW conditions was higher in DS (53.4 µg mg^−1^) compared to DT (20.3 µg mg^−1^) and increased in both cultivars under drought (by 3-fold and 4.5-fold, respectively; **Supplementary Figure S3B**). Similarly, cysteine content was low in both cultivars under the WW conditions and then increased significantly under drought (**Supplementary Figure S3C**).

To analyze the physiological impact of the drought treatments, we measured the maximum carboxylation rate (*V*_cmax_), electron transport rate (*J*), and mesophyll conductance (*g*_m_) in both cultivars. Mesophyll conductance decreased under drought stress and was not significantly different between the DS and DT cultivars (**Supplementary Figure S4B**). Meanwhile, *V*_cmax_ and *J* differed among the treatments but not between cultivars (**Supplementary Figures S4E, F**). However, the photosynthetic rates differed among the treatments and cultivars (**Supplementary Figure S4C**), but not in intrinsic WUE (**Supplementary Figure S4D**).

### Leaf epicuticular wax load and seed oil and protein content

**Figure 4:**
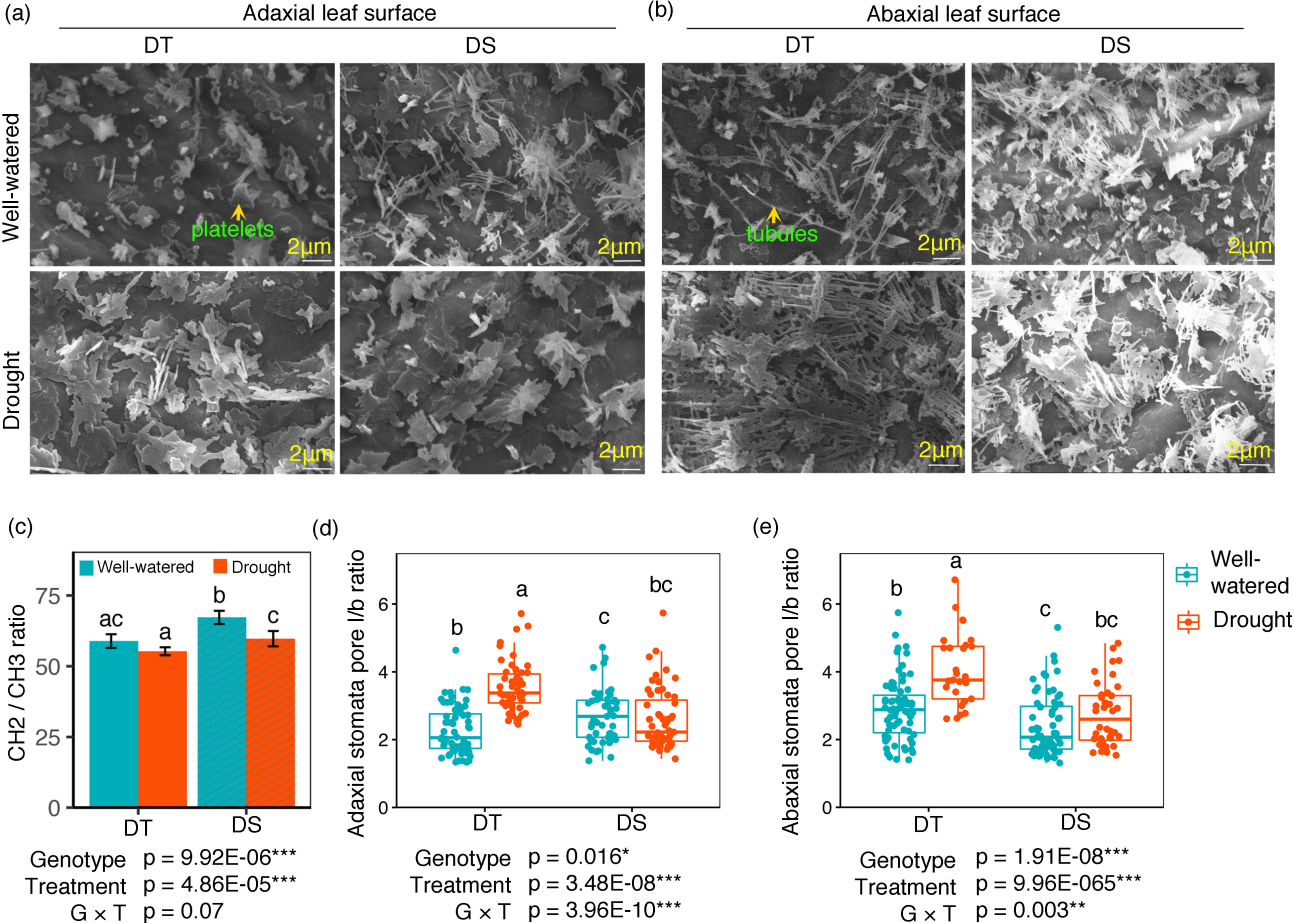
SEM morphology of adaxial **(A)** and abaxial **(B)** leaf surfaces from well-watered and drought canola cultivars. Leaf surface wax elements were detected at ×5,000 magnification (scale bars = 2 µm). The CH_2_/CH_3_ ratio **(C)**, stomatal pore opening represented as (l/b) ratio of pore length and width in adaxial **(D)** and abaxial **(E)** leaf surfaces for the drought-tolerant (DT) and drought-sensitive (DS) canola cultivars under well-watered and drought conditions. Data were analyzed using two-way ANOVA with *post-hoc* Tukey tests (letters indicate significant differences between groups at *p* < 0.05). Error bars depict standard deviation. Asterisks represent statistically significant differences between treatment and genotype (*p < 0.05, **p < 0.01, ***p < 0.001).

SEM images were taken for both the abaxial and adaxial surfaces of the leaves from plants under the WW and drought conditions. Wax morphology was different between treatments and to a lesser extent between the two cultivars (**Figures 4A, B**). The adaxial surface appeared to have more wax platelets, while the abaxial surface contained more wax tubules. Under drought, the DT canola cultivar developed more wax (platelets and *β*-diketone tubules).

Lipid unsaturation of the epicuticular waxes, measured by C=C content, decreased by 22.8% and 37.7%, for DT and DS, respectively, when plants were exposed to drought stress. Under this treatment, no significant difference was observed between the two cultivars (data not shown). The CH_2_/CH_3_ ratio had the same variation pattern as C=C with a higher value in the DS cultivar under the same treatment (**Figure 4C**). Drought treatment significantly reduced the CH_2_/CH_3_ ratio in the DS cultivar (**Figure 4C**). The C=O functional group increased by 30% under drought stress for the DS cultivar. However, there was no significant difference between the two cultivars (data not shown). We also measured the stomata pore length/breath (l/b) ratio of adaxial and abaxial surfaces in both cultivars under the WW and drought conditions. On both surfaces, the ratio increased significantly under drought treatment in the DT cultivar but not in the DS cultivar (**Figures 4D, E**).

Oil content (% seed dry matter) was similar between the DT and DS cultivars under the WW conditions (35.3% and 34.5%, respectively) (**Supplementary Table S1**). Under drought treatment, oil content decreased significantly to 23.8% and 23.7% for DT and DS, respectively. Similarly, seed protein content was not significantly different between DT and DS under the WW treatment (**Supplementary Table S1**). However, drought conditions significantly increased protein content in both cultivars.

### Metabolic analysis

Principal component analysis (PCA) of sucrose and organic acids was performed to provide a preliminary understanding of the metabolic differences between the DT and DS cultivars under the WW and drought conditions (**Supplementary Figure S5**). The PCA showed cultivar as the main factor for variance (37.5%) along PC1, while PC2 explained 30.1% of the variance.

**Figure 5:**
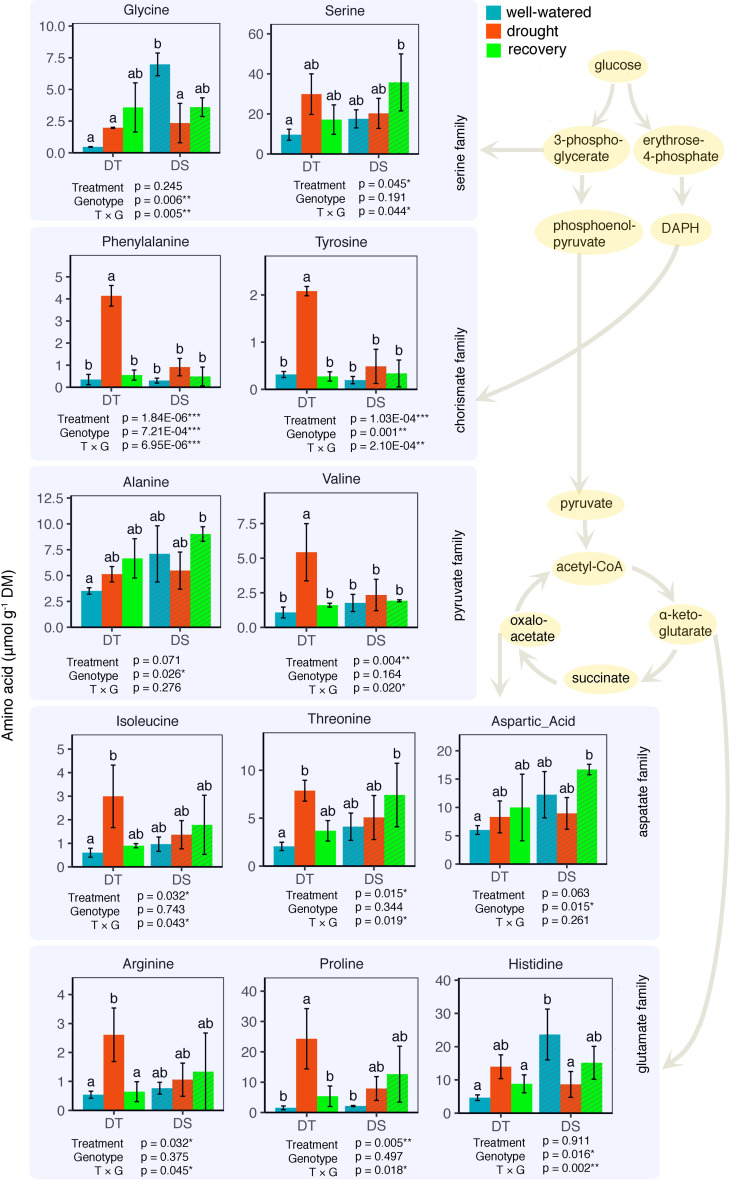
Leaf amino acid composition for the drought-tolerant (DT) and drought-sensitive (DS) canola cultivars under well-watered, drought, and water recovery conditions. Data were analyzed using two-way ANOVA with *post-hoc* Tukey tests (letters indicate significant differences between groups at *p* < 0.05). Error bars depict standard deviation. Amino acids are grouped based on different intermediates of the carbon metabolism pathway. Some intermediate components of the pathway are omitted for convenience.

**Figure 6:**
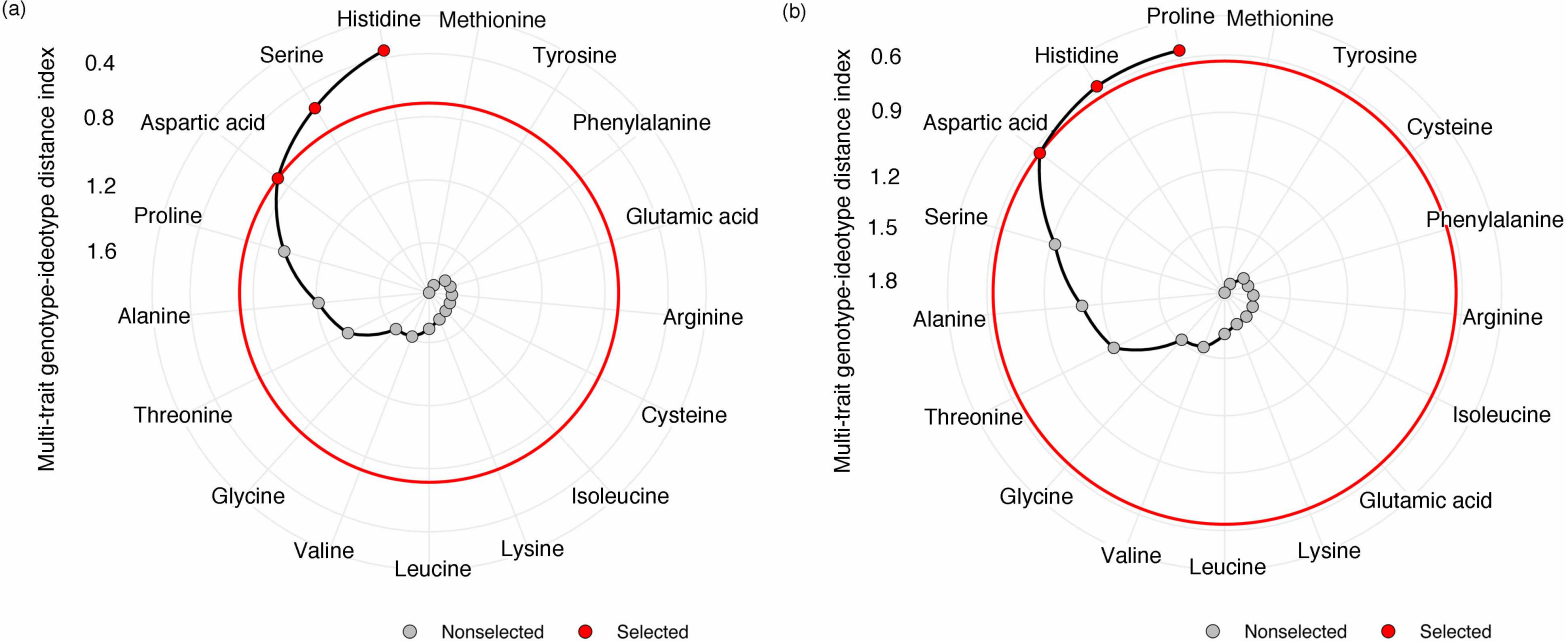
Amino acid ranking in ascending order for a multitrait genotype-ideotype distance index (MGIDI) from well-watered to drought conditions **(A)** and from drought to recovery **(B)**. The selected amino acids are shown in red circles.

**Figure 7:**
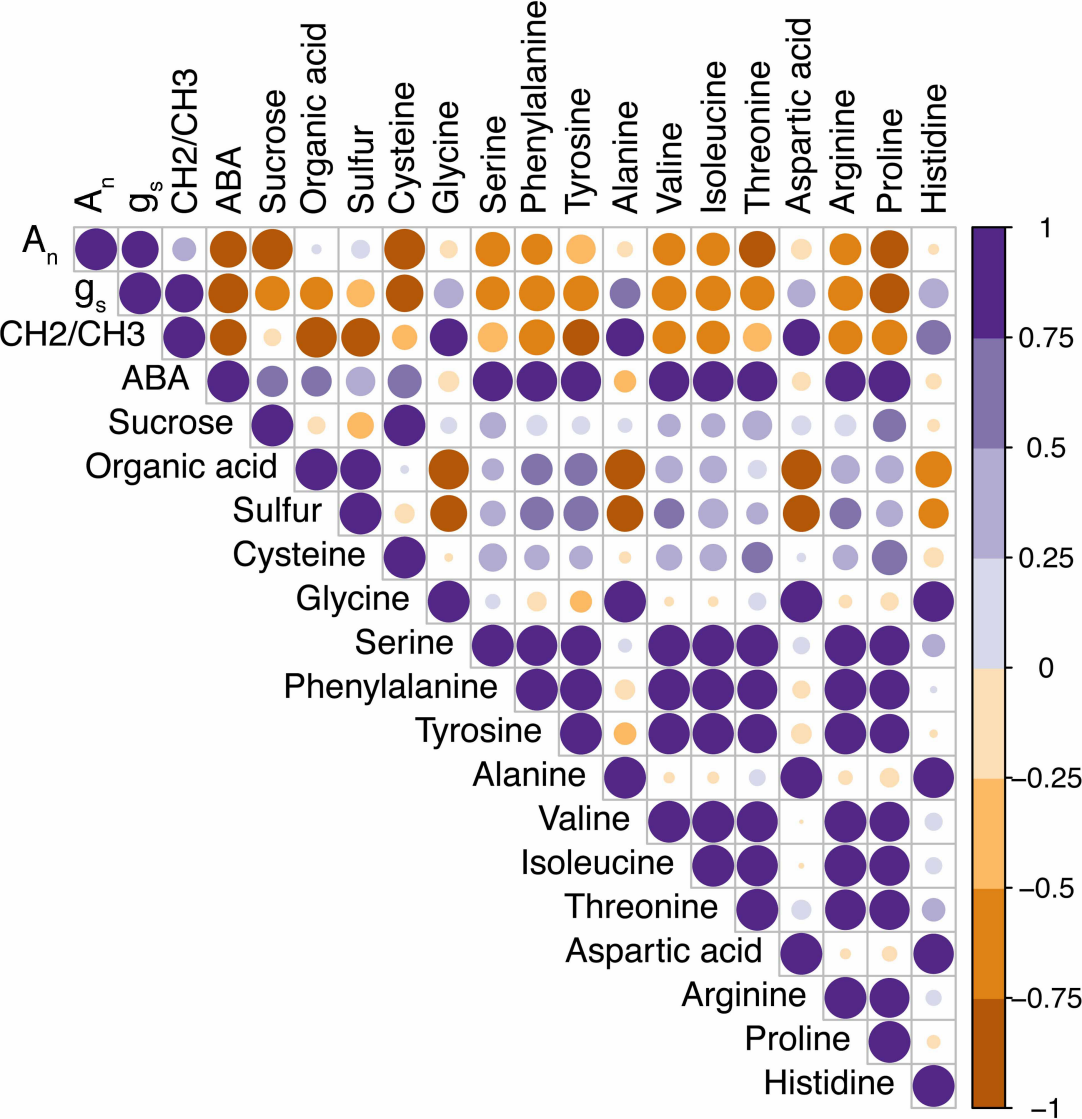
Correlation matrix of the physiological traits, wax load, and metabolites for drought-tolerant (DT) and drought-sensitive (DS) canola cultivars under well-watered and drought conditions. The increasing intensity of color and size of the bubble indicates positive (purple) and negative (brown) Pearson’s correlation.

**Figure 8:**
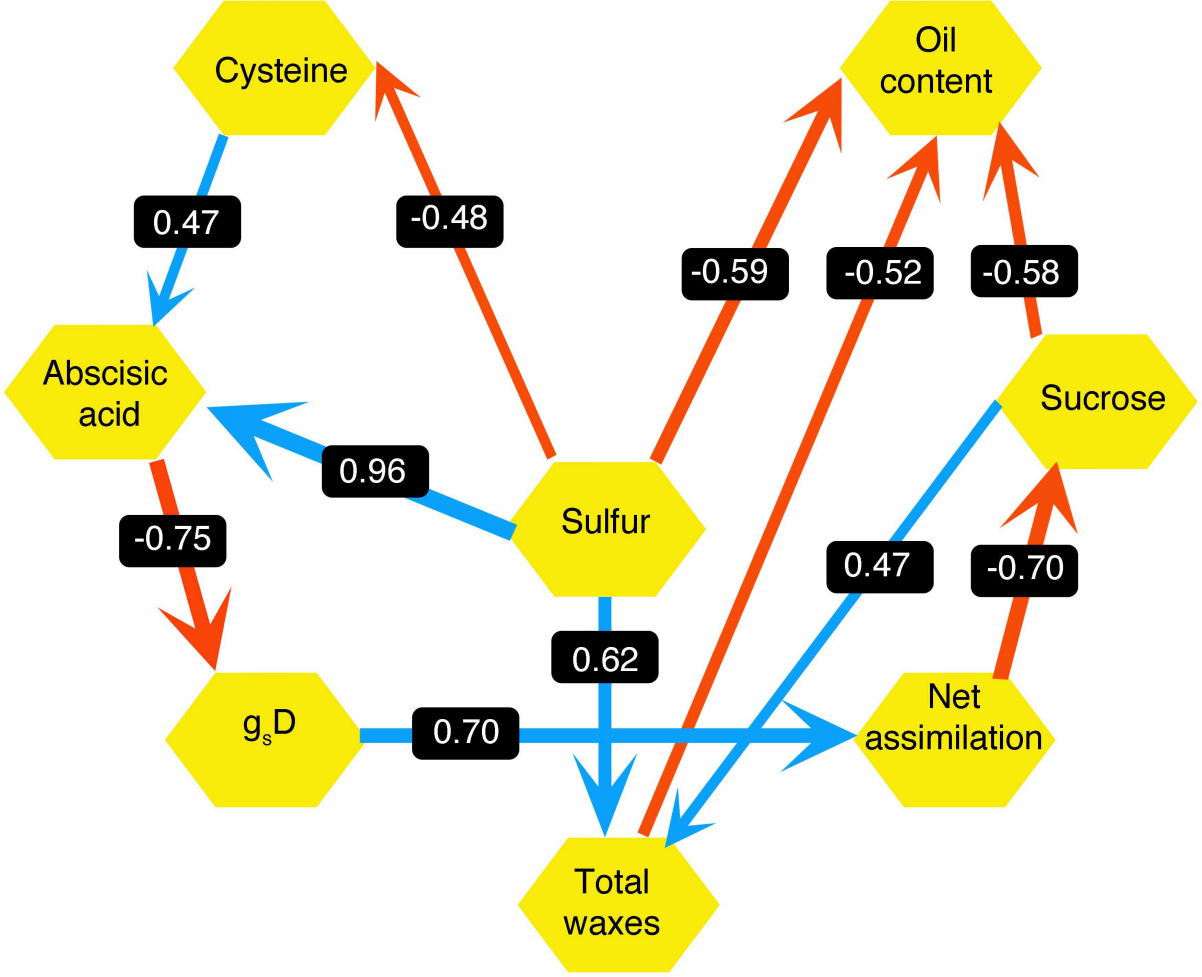
Pathway model hypothesized from agronomic, physiological, and metabolite traits for drought-tolerant (DT) and drought-sensitive (DS) canola cultivars under well-watered and drought conditions. Values on the arrow are path coefficients. Blue arrows indicate positive effects and red arrows indicate negative effects.

Next, we looked at how WW, drought, and drought recovery affected amino acids in both cultivars. We found that out of the 17 essential and non-essential amino acids analyzed, 12 of them had genotype and/or treatment × genotype effect. These 12 amino acids were grouped into five families based on their biosynthetic pathways from the intermediates of the carbon metabolism pathway (**Figure 5**). When we ranked these amino acids, MGIDI based on the WW and drought conditions selected histidine, serine, and aspartic acid (**Figure 6A**). Similarly, proline, histidine, and aspartic acid were selected when the drought and recovery selection criteria were applied (**Figure 6B**). A correlation matrix was plotted for physiological and metabolic variables that showed significant treatment or genotype effects (**Figure 7**). Lastly, we used the semPath function from semPlot for confirmatory factor analysis to demonstrate that leaf sulfur and sucrose content directly influenced oil content (**Figure 8**). The pathway diagram also revealed a clear connection between the production of cysteine, which is produced by diverting leaf sulfur, and the increase in ABA levels that results in stomatal closure.

## Discussion

Nocturnal stomatal conductance was considered a consequence of a non-complete control of stomata closing that leads to water leaking during nighttime (Resco de Dios et al., 2019). From a water balance perspective, this can be perceived as a loss that might compromise plant water status and growth. However, other explanations reporting a positive role of night transpiration have emerged, like the anticipation hypothesis stating that a higher predawn stomatal conductance is concomitant with a rapid opening of stomata and higher carbon assimilation at early morning (Resco de Dios et al., 2016). The decrease in water availability leads to stomatal closure to minimize leaf transpiration, which in turn results in a limited supply of CO_2_, reduced stomatal conductance, and decreased photosynthetic rate. In our study, under optimal conditions (the absence of water deficit), night *g*_s_ was lower in the drought-tolerant cultivar with no gain in yield (oil content in % of DM). However, under drought conditions, night *g*_s_ decreased but was not significantly different between the two cultivars. Then, though night *g*_s_ was genotype-specific and responsive to drought in canola, no evidence of a relationship with yield could be demonstrated under our experimental conditions. Then again, early morning and day stomatal conductance were both higher for the drought-sensitive cultivar under optimal and stressful water status of the plants, showing that the anticipation hypothesis could not be confirmed under our experimental setup. Several studies have demonstrated varying responses in the photosynthetic attributes of crops under stress, though these differences were not consistently linked to drought tolerance or reduced yield loss (Liu et al., 2018; Shariatipour et al., 2023). On the other hand, water loss also occurs through the leaf cuticle, which is a continuous lipid barrier, but not completely impermeable to water evaporation (Heredia-Guerrero et al., 2014). Indeed, epicuticular wax load and composition affect the whole water transpiration rate and are responsive to environmental cues (Domínguez et al., 2011; Lü et al., 2012). Total wax load of the leaves that we measured did not distinguish the two cultivars under optimal watering conditions. Genetic factors influence wax quantity and composition as well (Laila et al., 2017), but this seems to be triggered by the genotype × environment interaction in our case, rather than genotype alone. Actually, in *Brassica* spp., high wax load and low wax load genotypes could be distinguished within the same species (Laila et al., 2017; Jin et al., 2020). Our results showed that the drought-sensitive cultivar, unlike the drought-tolerant, was responsive to drought stress by increasing the total wax load, suggesting a genotype × treatment-driven response. Epicuticle molecules measured by FTIR were all influenced by exposure to the drought treatment, but only CH_2_/CH_3_ was affected by the genotype. In *Arabidopsis*, the analysis of the transcriptome showed that metabolism of leaf epicuticular waxes was upregulated by exposure to the soil water deficit (Bernard and Joubès, 2013). The observed variations of water loss through the stomata and cuticle showed that early morning and day stomatal conductance were indicators of drought tolerance among the two cultivars rather than EW load and structure, and night stomatal conductance, which were predominately affected by the environment (drought treatment).

Leaf ABA increased by water deficit and was higher in the DT cultivar. This is consistent with the variation of day stomatal conductance, significantly lower in DT and the strong negative correlation between *g*_s_ and ABA. This agrees with the ABA-mediated stomatal closure that has been observed in many species under drought and salinity stress (Christmann et al., 2007; Cutler et al., 2010). ABA is considered a good target to improve tolerance to drought since biosynthesis pathways, signaling, and regulation are now much better understood and the action on guard cells and stomata are well documented among staple crops (Wan et al., 2009; Vishwakarma et al., 2017). ABA-mediated closure of the stomata (through cytosol alkalization or Ca^2+^ release in the cytosol) might be directly triggered by soil water deficit detected in the roots or mediated by other signaling molecules through various biosynthesis cascades like that of hydrogen disulfide (H_2_S) (Finkelstein, 2013; Thakur and Anand, 2021). Our results also showed a positive relationship between leaf cysteine, a sulfur amino acid, and ABA content. Previous studies highlighted the importance of sulfur in mitigating the effect of stress in plants and showed that cysteine and other S derivatives are involved in signaling and in enhancing antioxidative response to abiotic stressors (Cao et al., 2014; Ma et al., 2016). Also, there is clear evidence of the interactions between S and other metabolites involved in stress tolerance like phytohormones, hydrogen peroxide (H_2_O_2_), and polyamines. In *Arabidopsis*, sulfate can induce stomata closure under water stress by activating NADPH oxidase to produce reactive oxygen species (ROS) that act as additional messengers of ABA signaling (Batool et al., 2018). *Arabidopsis* mutants lacking chloroplast sulfate transporter3;1 function (sultr3;1) showed lower ABA levels in the seeds and seedlings (Cao et al., 2014). Furthermore, studies reported that H_2_S, a signaling gas molecule produced by degradation of L-cysteine by L-cysteine desulfhydrase, was associated with ABA-dependent closure of stomata under biotic (pathogens) and abiotic stresses (oxidative, heavy metals) (Zhang et al., 2010; Sun et al., 2013; Pantaleno et al., 2021). The strong relation between ABA, sulfur, and cysteine demonstrated by the path analysis diagram of our work is consistent with these observations.

Yadav et al. (2019) found that certain amino acids are strong predictors of drought tolerance; specifically, a combination of three or four amino acids measured in greenhouse experiments can reliably predict yield traits in field conditions. High photorespiration during a drought increases serine’s availability, which is the building block of cysteine (Abadie and Tcherkez, 2019). It is evident from our data that drought treatment has a positive effect on sulfur assimilation and *de-novo* cysteine synthesis. The buildup of elevated aspartic acid levels along with other amino acids, such as proline, is significant during periods of drought and recovery phase as this mechanism may be advantageous in preventing leaf death and preserving carbon and nitrogen (Hayat et al., 2012). Canola plants may benefit from their leaves’ ability to withstand brief periods of drought because, once favorable moisture conditions return, photosynthetic activity can resume before new leaves emerge. Furthermore, aspartic acid assimilates inorganic nitrogen, giving plants a source of nitrogen for the synthesis of other nitrogen-containing compounds (Buchanan, 1980). One could speculate that exogenous aspartic acid application may enhance the endurance of cool-season canola by activating multiple metabolic pathways involved in the drought adaptation of plants. Largely, aspartic acid has the potential to function as a drought-responsive biomarker due to the rapid changes in concentration it experiences in stressed plants. At the same time, phenylalanine, a precursor for the biosynthesis of several antioxidants (CAT, SOD, and POD), increased in canola plants grown under drought stress, especially in the drought-tolerant cultivar (Ramzan et al., 2023). In fact, antioxidant activity was found to be closely linked to flavonoids. Under drought stress, flavonoids such as myricetin and epicatechin showed increased accumulation in rapeseed (Salami et al., 2024a). Higher levels of antioxidants and total flavonoid were also reported in several accessions of Kentucky bluegrass under drought stress (Shariatipour et al., 2022).

Under drought, La et al. (2019) reported an accumulation of sugars, particularly sucrose, due in part to the high expression of ABA-dependent sucrose signaling genes in *Brassica napus*. In rice, Mathan et al. (2021) showed that an ABA‐responsive transcription factor, OsbZIP72, directly binds to the promoters of two sugar transporters (OsSWEET13 and OsSWEET15) and activates their expression. This increase in sucrose content was observed in our study for both cultivars with a higher content in the drought-sensitive cultivar. However, we could not observe a significant correlation between ABA and sucrose as shown in **Figure 7**.

Seed oil content decreased while protein content increased in both cultivars under drought treatment, but this did not differentiate the two cultivars. In our previous study, Elferjani and Soolanayakanahally (2018) showed a similar increase in seed protein content while oil content was not significantly different between the control and water-stressed plants. Other studies reported a decrease in oil content by soil water deficit (Moaveni et al., 2010; Tesfamariam et al., 2010; Hatzig et al., 2018). These different responses might be explained by cultivar-specific responses of oil and protein content to drought as reported in the study of Hatzig et al. (2018) on a group of eight canola cultivars. Similarly, Guo et al. (2017) showed a significant genotype × environment effect on seed oil content of nine semiwinter rapeseed lines and their 72 F_1_ hybrids. Accumulation of triacylglycerols, the main components of canola oil, might be influenced by the environment at every step of their biosynthesis (Mácová et al., 2022). For example, oil content is dependent on silique wall photosynthates, which are transported to the seed coat and transformed into fatty acids (Baud and Lepiniec, 2010). Then, drought, which might affect silique development and growth, would consequently decrease the photosynthates produced and ultimately oil content (Ghobadi et al., 2006; Naderi and Emam, 2010). However, along with the availability of photoassimilates, oil content also depends on the seed intrinsic capacity for oil accumulation, particularly controlled by the embryo and genotype × environment interactions (Weselake et al., 2009).

## Conclusions

In conclusion, our study shows a noticeable response of leaf sulfur and cysteine to drought exposure in both canola cultivars, which agrees with previous studies that demonstrated the role of sulfur and its derivatives in mitigating the effects of abiotic stressors on plants. The correlations between cysteine, ABA, and stomatal conductance suggest a role of sulfur and cysteine in modulating the stomata movement under water deficit conditions. Our study also demonstrated the responsiveness of the load and composition of epicuticular waxes to drought in canola and the importance of water losses through the stomata during the dark period and early morning. Attributes related to CO_2_ diffusion and photosynthetic capacity were significantly different between the two cultivars and could indicate a higher drought tolerance for the DT cultivar. A combination of three amino acids (phenylalanine, aspartic acid, and serine) may be a more accurate predictor of drought tolerance in canola than an individual amino acid.

## Data Availability

The raw data supporting the conclusions of this article will be made available by the authors, without undue reservation.

## Supplementary material

Supplementary Figure 1 Various agronomic traits for drought-tolerant (DT) and drought-sensitive (DS) canola cultivars under well-watered and drought conditions; plant height **(A)**, normalized difference vegetation index (NDVI) **(B)**, days to flowering **(C)**, number of branches **(D)**, seed yield **(E)** and seed yield per pod **(F)**. Data was analyzed using two-way ANOVA with *post hoc* Tukey tests (letters indicate significant differences between groups at p < 0.05. Error bars depict standard deviation. Each value represents the mean ± SD and the asterisks represent statistically significant differences between Genotype (G) and Treatment (T) (**p* < 0.05, ***p* < 0.01, ****p* < 0.001).

Supplementary Figure 2 Leaf temperature obtained from thermal images between 0800h to 1800h for **(A)** drought-tolerant (DT) and **(B)** drought-sensitive (DS) canola cultivars under well-watered and drought conditions. Each value represents the mean ± SE and the asterisks represent statistically significant differences between treatments (ns= not significant, **p* < 0.05, ***p* < 0.01, ****p* < 0.001, *****p* < 0.0001).

Supplementary Figure 3 Leaf ABA **(A)**, sucrose **(B)** and cysteine **(C)** contents for the drought tolerant (DT) and drought sensitive (DS) cultivars under well-watered and drought conditions. Data was analyzed using two-way ANOVA with *post hoc* Tukey tests (letters indicate significant differences between groups at p < 0.05. Error bars depict standard deviation. Asterisks represent statistically significant differences between treatment (T) and Genotype (G) (**p* < 0.05, ***p* < 0.01, ****p* < 0.001).

Supplementary Figure 4 Physiological traits for the drought tolerant (DT) and drought sensitive (DS) cultivars under well-watered and drought conditions. Data was analyzed using two-way ANOVA with *post hoc* Tukey tests (letters indicate significant differences between groups at p < 0.05. Error bars depict standard deviation. Numbers in (g) are p values and asterisks represent statistically significant differences between treatments (T) and genotypes (G) (**p* < 0.05, ***p* < 0.01, ****p* < 0.001).

Supplementary Figure 5 Principal component analysis of drought-tolerant (DT) and drought-sensitive (DS) canola cultivars under well-watered and drought conditions using organic acids and sucrose values. X and Y axis show principal component 1 and principal component 2 that explain 37.5% and 30.1% of the total variance, respectively. A dotted eclipse represents a 95% confidence level.
