# Supplementary figures and images for: Drought induced metabolic shifts and water loss mechanisms in canola: role of cysteine, phenylalanine and aspartic acid
Source: Front Plant Sci. 2024 Dec 23;15:1385414. doi: 10.3389/fpls.2024.1385414 (PMC11707614; doi:10.3389/fpls.2024.1385414)

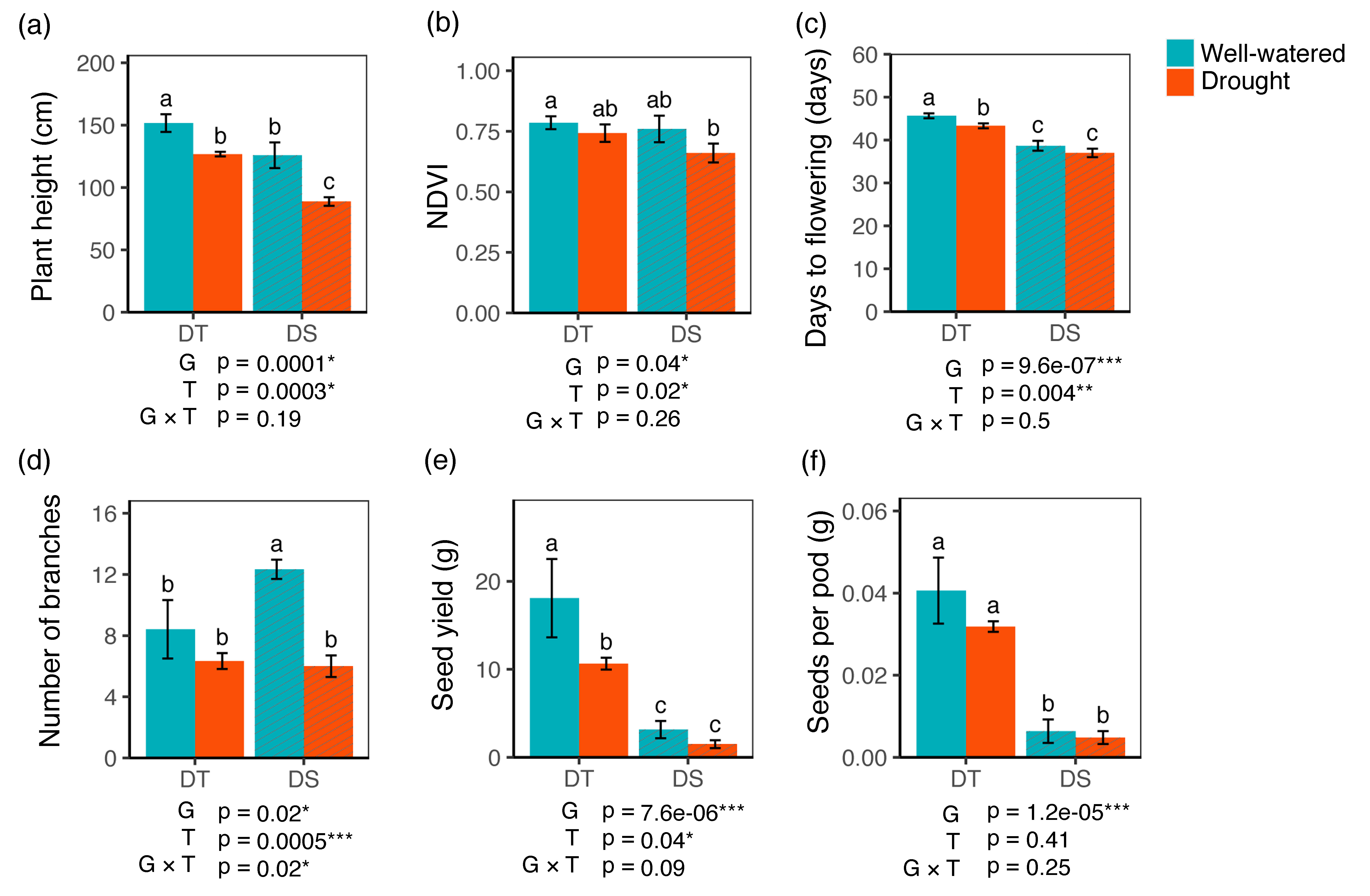

Supplement: Supplementary Figure 1 — Various agronomic traits for drought-tolerant (DT) and drought-sensitive (DS) canola cultivars under well-watered and drought conditions; plant height (A), normalized difference vegetation index (NDVI) (B), days to flowering (C), number of branches (D), seed yield (E) and seed yield per pod (F). Data was analyzed using two-way ANOVA with post hoc Tukey tests (letters indicate significant differences between groups at p < 0.05. Error bars depict standard deviation. Each value represents the mean ± SD and the asterisks represent statistically significant differences between Genotype (G) and Treatment (T) (*p < 0.05, **p < 0.01, ***p < 0.001). [file Image1.jpg]

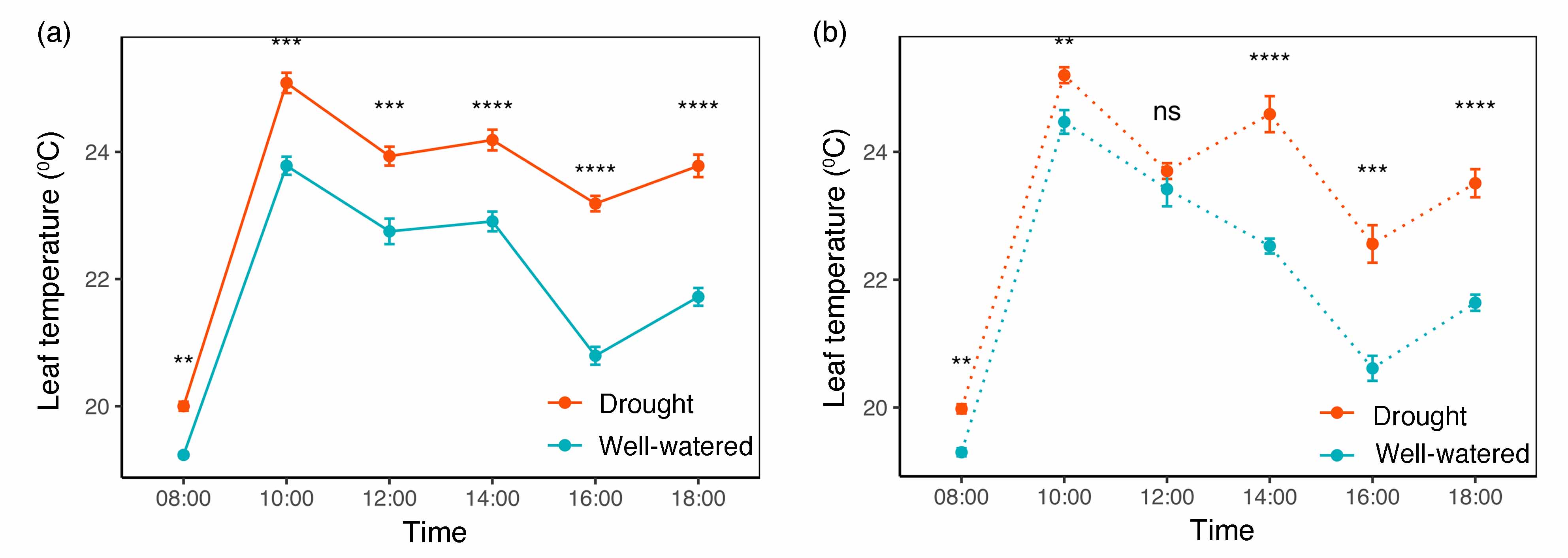

Supplement: Supplementary Figure 2 — Leaf temperature obtained from thermal images between 0800h to 1800h for (A) drought-tolerant (DT) and (B) drought-sensitive (DS) canola cultivars under well-watered and drought conditions. Each value represents the mean ± SE and the asterisks represent statistically significant differences between treatments (ns= not significant, *p < 0.05, **p < 0.01, ***p < 0.001, ****p < 0.0001). [file Image2.jpg]

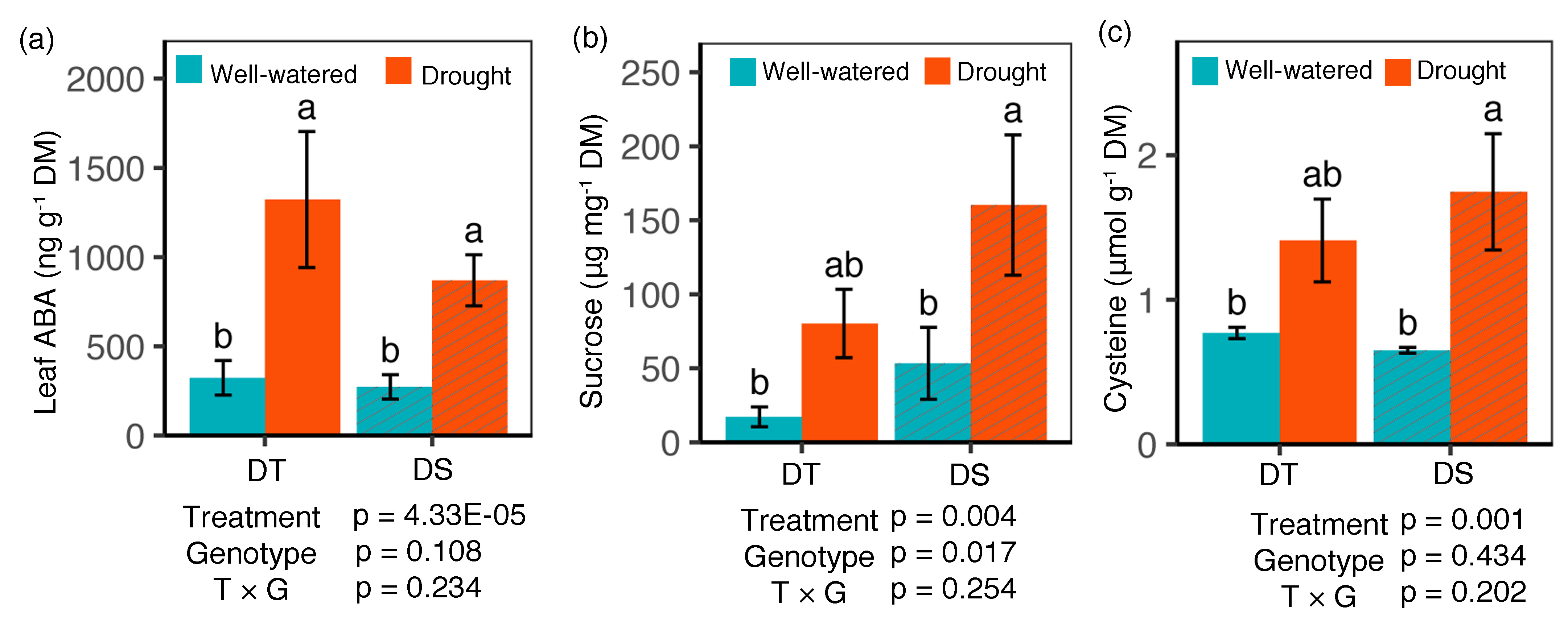

Supplement: Supplementary Figure 3 — Leaf ABA (A), sucrose (B) and cysteine (C) contents for the drought tolerant (DT) and drought sensitive (DS) cultivars under well-watered and drought conditions. Data was analyzed using two-way ANOVA with post hoc Tukey tests (letters indicate significant differences between groups at p < 0.05. Error bars depict standard deviation. Asterisks represent statistically significant differences between treatment (T) and Genotype (G) (*p < 0.05, **p < 0.01, ***p < 0.001). [file Image3.jpg]

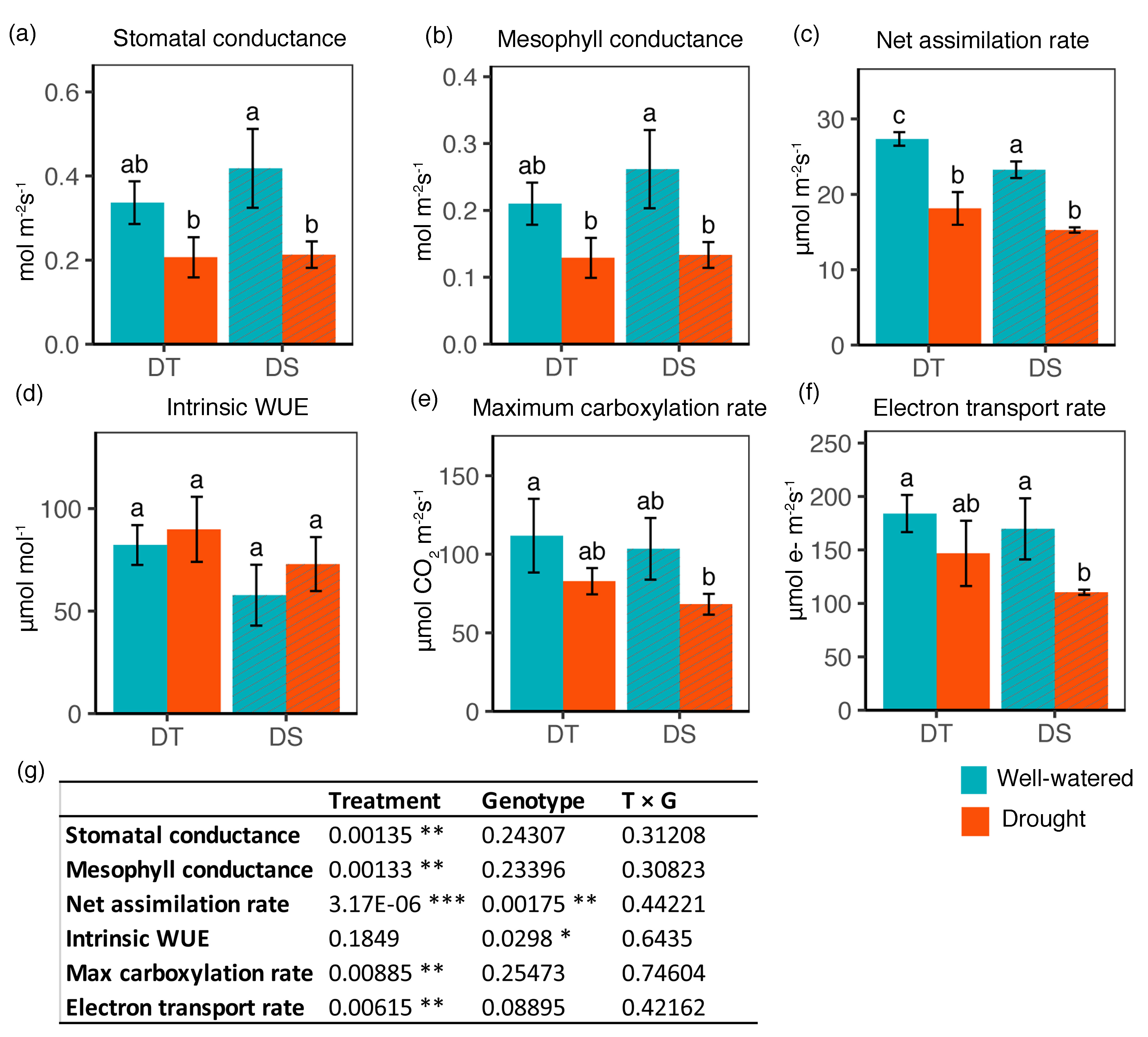

Supplement: Supplementary Figure 4 — Physiological traits for the drought tolerant (DT) and drought sensitive (DS) cultivars under well-watered and drought conditions. Data was analyzed using two-way ANOVA with post hoc Tukey tests (letters indicate significant differences between groups at p < 0.05. Error bars depict standard deviation. Numbers in (g) are p values and asterisks represent statistically significant differences between treatments (T) and genotypes (G) (*p < 0.05, **p < 0.01, ***p < 0.001). [file Image4.jpg]

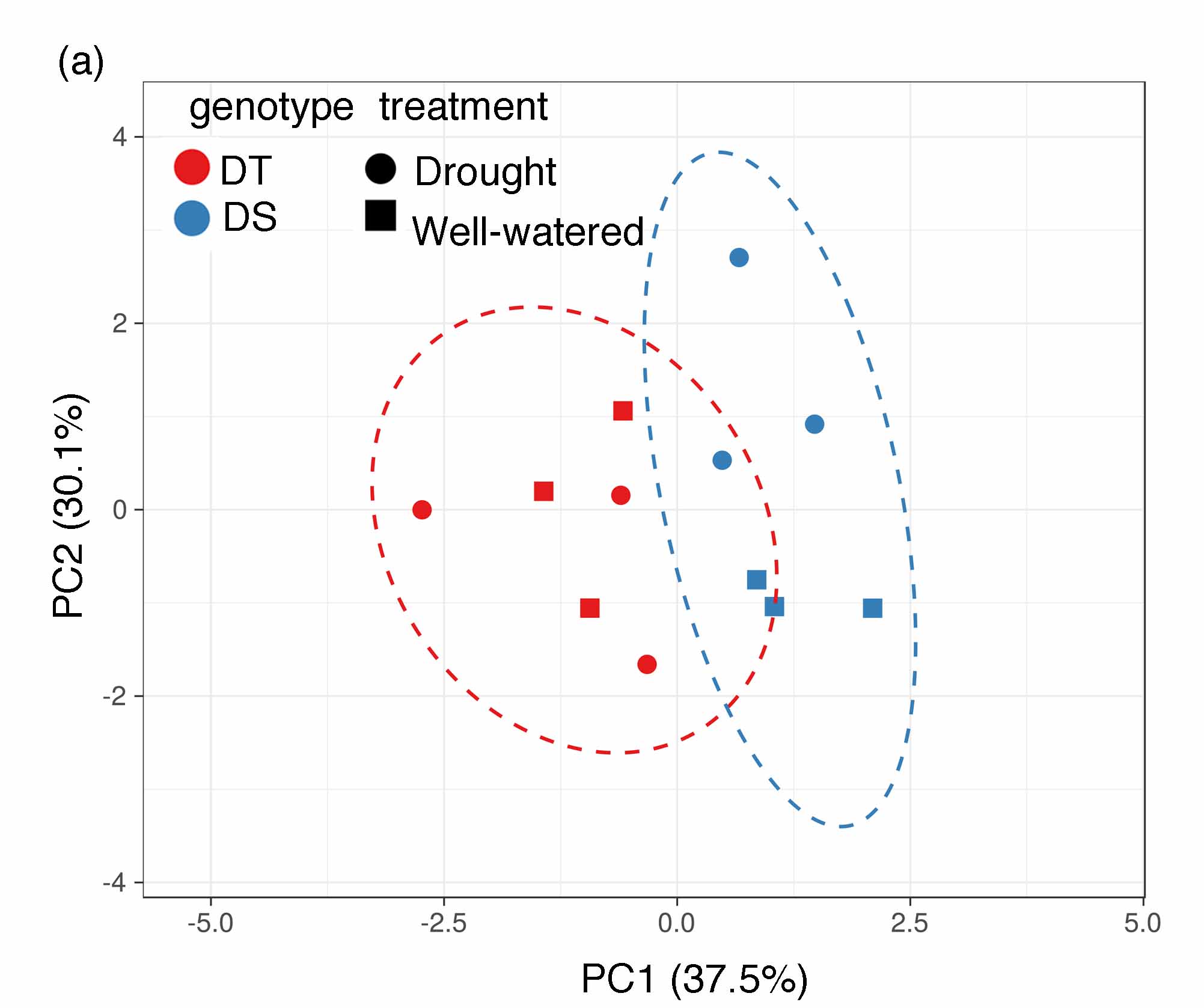

Supplement: Supplementary Figure 5 — Principal component analysis of drought-tolerant (DT) and drought-sensitive (DS) canola cultivars under well-watered and drought conditions using organic acids and sucrose values. X and Y axis show principal component 1 and principal component 2 that explain 37.5% and 30.1% of the total variance, respectively. A dotted eclipse represents a 95% confidence level. [file Image5.jpg]
