# Supplementary material for: Drought induced metabolic shifts and water loss mechanisms in canola: role of cysteine, phenylalanine and aspartic acid
Source: Front Plant Sci. 2024 Dec 23;15:1385414. doi: 10.3389/fpls.2024.1385414 (PMC11707614; doi:10.3389/fpls.2024.1385414)
Supplement: Supplementary file 6 [file Table1.docx]

**Drought-Induced Metabolic Shifts and Water Loss Mechanisms in Canola: Role of Cysteine Phenylalanine and Aspartic Acid**

Read Elferjani^1^, Shankar Pahari^1^, Raju Soolanayakanahally^1*^, Krista Ballantyne^1^, Eiji Nambara^2^

**Supplementary information**

**Table S1**: Seed oil and seed protein content in DT and DS cultivars under well-watered and drought treatment.

|  | DT | | DS | |
| --- | --- | --- | --- | --- |
|  | WW | D | WW | D |
| Seed oil (%) | 35.3 ^a^ ± 0.30 | 23.8 ^b^ ± 1.7 | 34.5 ^a^ ± 0.85 | 23.7 ^b^ ± 2.55 |
| Seed protein (%) | 32.2 ^a^ ± 0.57 | 38.5 ^b^ ± 1.02 | 33.2 ^a^ ± 0.64 | 38.4 ^b^ ± 0.16 |
